# Flexible Polymer-Ionic Liquid Films for Supercapacitor Applications

**DOI:** 10.3390/gels9040338

**Published:** 2023-04-16

**Authors:** Christo Novakov, Radostina Kalinova, Svetlana Veleva, Filip Ublekov, Ivaylo Dimitrov, Antonia Stoyanova

**Affiliations:** 1 Institute of Polymers, Bulgarian Academy of Sciences, Sofia, Acad. G. Bonchev Str., 103, 1113 Sofia, Bulgaria; 2 Institute of Electrochemistry and Energy Systems, Bulgarian Academy of Sciences, Acad. G. Bonchev Str., 10, 1113 Sofia, Bulgaria

**Keywords:** ionic liquid, conductive polymer film, gel polymer electrolyte, cross-linked separator, electrochemical tests, supercapacitor cell

## Abstract

Mechanically and thermally stable novel gel polymer electrolytes (GPEs) have been prepared and applied in supercapacitor cells. Quasi-solid and flexible films were prepared by solution casting technique and formulated by immobilization of ionic liquids (ILs) differing in their aggregate state. A crosslinking agent and a radical initiator were added to further stabilize them. The physicochemical characteristics of the obtained crosslinked films show that the realized cross-linked structure contributes to their improved mechanical and thermal stability, as well as an order of magnitude higher conductivity than that of the non-crosslinked ones. The obtained GPEs were electrochemically tested as separator in symmetric and hybrid supercapacitor cells and showed good and stable performance in the investigated systems. The crosslinked film is suitable for use as both separator and electrolyte and is promising for the development of high-temperature solid-state supercapacitors with improved capacitance characteristics.

## 1. Introduction

In order to improve the conductivity of polymers, determining their use as materials for energy storage devices, the inclusion of ionic liquids (ILs) in their preparation has been widely used in recent years. Polymer films thus obtained have been applied by a number of researchers of supercapacitors. Despite the fact that very high capacity has not yet been achieved and data on their stability in most cases are lacking, they are promising for practical applications, including at high temperature. Therefore, a major research interest is focused on improving the specific energy of these devices.

The preparation of conductive films by the well-known solution casting technique has been used by a number of authors via the co-dissolution of a matrix forming polymer—most often poly(vinylidene fluoride-co-hexafluoropropylene) (P(VDF-HFP)) and a doping ionic salt or ionic liquid [1,2,3,4].

Gel polymer electrolyte (GPE) film prepared by mixing P(VDF-HFP) plasticizers (ethylene- and propylene carbonate) and doping salt—tetraethylammonium tetrafluoroborate (TEABF_4_)—was used in an electrical double-layer capacitor (EDLC) [5]. A composite polymer electrolyte based on a P(VDF-HFP) matrix containing LiPF3 (CF_3_CF_2_)_3_ electrolyte salt mixed with plasticizers and nanoscopic Al_2_O_3_ as a filler exhibiting ionic conductivity of 0.98 mScm^−1^ was also proposed [6]. Another example of application of thin solid polymer electrolyte film for lithium-ion batteries describes the preparation of P(VDF-HFP)/LiBF_4_ membrane. The ionic conductivity increased with the variation of salt concentration. The membrane containing 40 wt% of salt has the highest ionic conductivity, reaching a value of 1.965 mScm^−1^. Further increase in salt concentration to 50 wt% reduces the mobility of charge carriers and the ionic conductivity decreases as it has been observed. [7].

High conductivity P(VDF-HFP) films doped with 1-ethyl-3-methylimidazolium thiocyanate ionic liquid have been prepared. The EDLC fabricated using the film with the highest conductivity (80 wt% IL) showed a specific capacitance of 2.36 Fg^−1^ [4].

Considering various advantages of solid/quasi-solid electrolytes over liquid electrolytes, a number of groups have tested the performance of supercapacitor cells with GPEs containing different redox-additives [8,9,10]. A non-aqueous GPE comprising an ionic liquid 1-ethyl-3-methylimidazolium bis(trifluoromethylsulfonyl)imide with potassium iodide as an additive, entrapped in P(VDF-HFP), is presented and evaluated for application in carbon quasi-solid-state-supercapacitors. The incorporation of redox-additive in GPE has been found to improve the capacitance, energy and the cycle life of supercapacitors [11,12].

Back in 2004, Wang et al. prepared cross-linked PVDF/poly(ethylene glycol) dimethacrylate (PEGDMA) microporous membranes without ionic liquid. The ionic conductivity was achieved by immersing the membranes in a liquid electrolyte. Test supercapacitor cells were subsequently formed by sandwiching the membrane between two stainless steel electrodes [13].

By incorporation of IL (EMIBF_4_) into a PEGDMA matrix a quasi-solid-state electrolyte was prepared for high-temperature application of supercapacitors [14].

Recently we developed a hybrid supercapacitor with improved characteristics by introducing P(VDF-co-HFP) as an effective polymer binder in the mass of the active electrode. The copolymer favors the wettability of the electrode while smoothing its surface; thus, ensuring easier penetration of IL into the pores of the carbon material, significantly improving the electrochemical characteristics of the studied systems [15].

In this study, we aimed to evaluate the possibilities for application of stabilized GPE films based on crosslinked P(VDF-co-HFP)/poly(ethylene glycol) diacrylate (PEGDA) polymer matrix with immobilized different ionic liquids (in solid or liquid aggregate state) both as separator and electrolyte in a supercapacitor cell. The cross-linking was carried out in the presence of an oligomeric diacrylate reagent and a radical initiator at elevated temperature after slow solvent evaporation and film deposition. Both non-stabilized as well as crosslinked GPE films were structurally and thermally characterized. Finally, the supercapacitor cells assembled with non-stabilized and crosslinked GPE films were investigated and their electrochemical parameters were compared.

## 2. Results and Discussion

Gel polymer electrolytes (GPEs) fabricated by immobilization of ionic liquids (ILs) in suitable host polymers are of growing interest as potential materials for supercapacitor applications due to their various advantageous merits, i.e., non-volatility and non-flammability as well as enhanced mechanical stability due to their plasticizing properties.

As already noted in the introduction, few examples of films obtained from P(VDF-HFP) and ionic liquid using the cited solution casting methodology for electrochemical applications are described in the literature. The use of an EMIBF_4_-doped cross-linked film with a PEGDMA matrix has been proposed [14]. There is also an example of obtaining a P(VDF-HFP) film cross-linked in the presence of a linker that does not contain ionic liquid [13]. To the best of our knowledge, there are no examples in the literature for the use of crosslinked-stabilized films doped with our proposed ionic liquids for supercapacitor cell applications.

**Figure 1:**
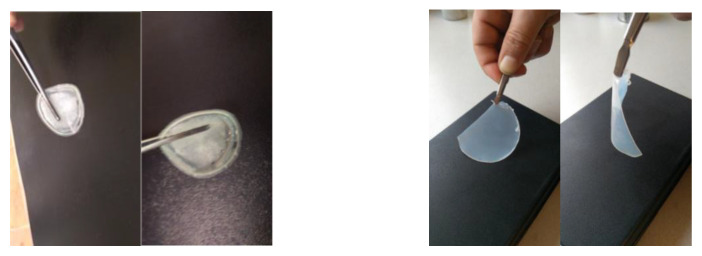
Non-stabilized P(VDF-HFP/IL-L) (**left**) and cross-linked P(VDF-HFP/PEGDA/IL-L) films (**right**).

Non-aqueous, quasi-solid GPE flexible films have been formulated by immobilization of ILs, namely, 1-ethyl-3-methylimidazolium iodide or 1-buthyl-1-methylpyrrolidinium bis(trifloromethylsulfonyl)imide in a host poly(vinylidenefluoride-co-hexafluoropropylene) (P(VDF-HFP)) (Figure 1 left). Quasi-solid films with different content of ionic liquid were obtained. The maximum possible amount of IL in liquid state, which can be used to obtain a stable film in composites with the selected polymer, has been established.

The obtained cross-linked films (P(VDF-HFP)/IL-S (L)) (Figure 1 right) are much more stable exhibiting excellent conductivity (see below). They do not lose content of the incorporated ionic liquid, and possess good mechanical properties—much better flexibility and stability when subjected to electrochemical tests, compared to their unstabilized (non-cross-linked) counterparts.

All GPE composite membranes have been thermally, structurally and electrochemically characterized.

The polymer-ionic liquid films have been characterized by FTIR spectroscopic analysis to study possible interactions and complexation of the host polymer matrix with ILs.

Table S1 and Figure S1 available in the Supplementary Materials section show the IR spectra peak positions and their assignments for pure P(VDF-HFP) and IL-L electrolyte. The vibrational bands at 489, 613, 762, 795, 840,872, 974, 1066 and 1181 cm^−1^ are common to the host polymer P(VDF-HFP) and correspond to a semi-crystalline nature of the polymer [1,16].

It must be noted that the spectrum of pure P(VDF-HFP) membrane exhibits much fewer absorption bands in the range between 520 and 800 cm^−1^ and almost no absorptions between 1300 and 1380 cm^−1^, and above 1400 cm^−1^ where one can find some of the absorptions that are characteristic for the pure IL. The mid-infrared spectral region of IL between 500 and 1500 cm^−1^ shows distinct peaks of the various CF_3_, SNS and SO_2_ stretching modes from the TFSI anion which are consistent with the literature [17].

The interaction between the polymer chain and IL generates changes in the chemical structure and physical properties which in turn influences vibrational modes of atoms and molecules and eventually causes changes in the corresponding spectrum. Some noticeable changes are observed in the spectrum upon mixing the polymer and IL and formulating polymer-IL films confirming interactions and complexation of IL with the host polymer matrix. Upon adding the IL, the bands of P(VDF-HFP) at 489 cm^−1^ corresponding to CF_2_ bending and wagging and at 762 cm^−1^ (CH_2_ rocking) become of significantly reduced intensity, while bands at 974 cm^−1^ (C-F stretching) and 1383 cm^−1^ (CH_2_ wagging) disappear, respectively. A few noticeable shifts in bands, namely, from 795 (CF_3_ stretching) to lower frequencies of 788 cm^−1^, from 872 (combined CF_2_ and CC symmetric stretching) to higher ones of 882 cm^−1^ and from 1066 (CF stretching) to 1053 cm^−1^ are also noted. All spectral changes indicate, as mentioned previously, the implementation of conformational changes in the polymer matrix during the process of polymer-ionic electrolyte membrane formation, as a result of interaction of IL species with the polymer chain [1,11].

**Figure 2:**
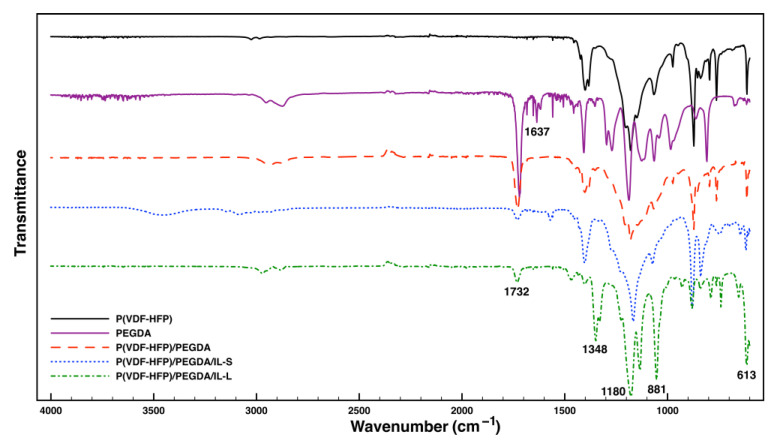
FTIR spectra of pure P(VDF-HFP), PEGDA and cross-linked P(VDF-HFP)/PEGDA; P(VDF-HFP)/PEGDA/IL-S (EMIMI); P(VDF-HFP)/PEGDA/IL-L (BMPTFSI).

The successful cross-linking process of pure P(VDF-HFP) film as well as membrane doped with ionic liquid in the presence of PEGDA and a radical initiator was also verified. The IR spectra of cross-linked P(VDF-HFP)/PEGDA/IL membranes (both with the ionic liquid in the liquid state and with that in the solid state, respectively), prove the successful progress of the polymerization process initiated with the selected initiator (Figure 2). The characteristic absorption bands of PEGDA are clearly visible in the IR spectrum of the cross-linking reagent (Figure 2). The absorption band at 1637 cm^−1^ may be attributed to the C=C aliphatic double bond, the band at 1720 cm^−1^ corresponds to the carbonyl C=O group, whereas the band at 1188 cm^−1^ corresponds to C-O-C from the polyether side groups. After the cross-linking reaction was performed on the membranes at elevated temperature, the band at 1637 cm^−1^ completely disappeared from the FTIR-spectra of the corresponding products, whereas the band attributed to the carbonyl groups is clearly visible. This is an indication that the cross-linking reaction preferentially proceeded to completion at the expense of the C=C bonds [18]. This is because the bond energy of C=C bonds is much lower, as compared to that of C=O bonds [19].

**Figure 3:**
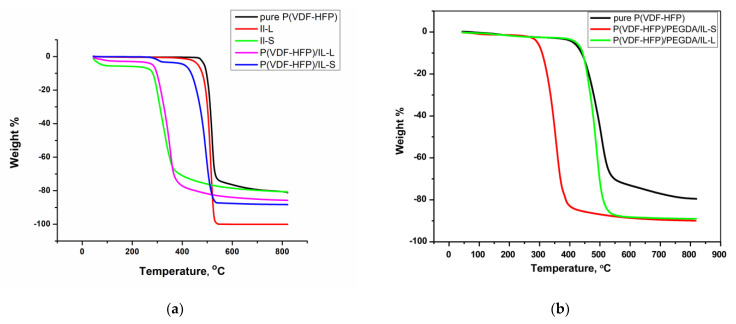
TGA thermograms of non-stabilized P(VDF-HFP), P(VDF-HFP)/IL-L (S) (**a**) and cross-linked P(VDF-HFP)/PEGDA/IL-L (S) (**b**) films.

The thermal stability range and possible phase transitions (glass transition, crystallization and melting temperature) of both non-stabilized and crosslinked polymer and polymer/ionic liquids membranes were tested using TGA and DSC analyses. Figure 3 illustrates the comparative TGA plots of non-stabilized P(VDF-HFP) films doped with solid IL-S or liquid IL-L (a) and of cross-linked membranes (b). As observed by other authors [1,11,14] the thermal stability of pure P(VDF-HFP) membrane is high with degradation starting at temperatures beyond 400 °C. The thermal stability of the ionic liquid-doped films is lower than that of the pure polymer matrix, implying a possible interaction of the ionic liquid (electrostatic involving both the anion and the counter ion) with the host polymer chain. As a rule, the decomposition proceeds predominantly in one stage. In the case of doping with the ionic liquid in the liquid phase and a redox additive of potassium iodide, an initial loss of about 4% mass is observed at about 250–270 °C, which can be attributed to moisture content due to the hygroscopic nature of the KI salt as suggested by others [11]. The thermal stability of the membrane containing the IL in liquid phase is greater than that of the solid IL one in the parent P(VDF-HFP) polymer matrix, which is due to the higher decomposition temperature of the IL-L. It should be noted that the thermal stability of the IL-L containing cross-linkages with PEGDA membrane is superior to that of the non-stabilized one, with the thermal profile completely following that of the pure polymer matrix. Therefore, the stronger incorporation of the ionic liquid into the network of the stabilized polymer membrane improves its thermal stability, simultaneously with the improvement of mechanical properties and conductivity.

**Figure 4:**
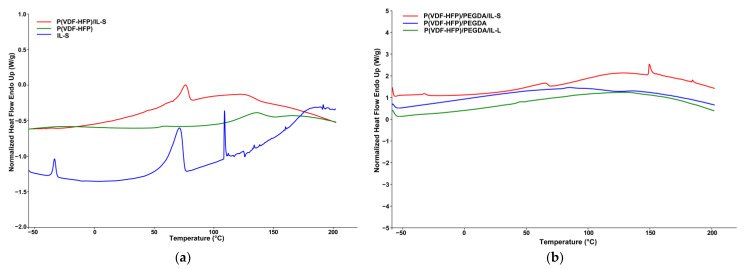
DSC curves of pure P(VDF-HFP), IL-S and P(VDF-HFP)/IL-S film (**a**) and cross-linked with PEGDA P(VDF-HFP) and P(VDF-HFP)/IL-L (S) (**b**).

DSC profiles of pure P(VDF-HFP), IL-S and P(VDF-HFP)/IL-S film recorded in a temperature range from −50 to 180 °C are presented in Figure 4a. The endothermic peak in the thermogram of film prepared from pure polymer at about 140 °C is attributed to the polymer melting temperature [20]. The profile of IL-S shows two endothermic peaks at ~-36 °C and at ~76 °C, respectively. The observation of a second endothermic transition may also be due to the presence of a second component in the commercial product. This may also be the reason for the observed first stage with about 5% weight loss in the TGA curve (see Figure 3a). In general, calorimetric measurements of ionic liquids may exhibit complicated patterns of phase transitions including crystallization, glass transition, solid-solid transitions and complex pre-melting behavior as it has been discussed in a number of papers [21,22,23,24,25,26]. In the thermogram of the film doped with IL, the temperature transitions characteristics of ionic liquid are observed. In addition, a noticeable decrease in peaks corresponding to the melting temperature of P(VDF-HFP) is observed. It can be speculated with certainty that the melting temperature has shifted downwards in sample P(VDF-HFP)/IL-S in comparison to the pure P(VDF-HFP) sample.

The DSC thermograms of cross-linked membranes presented in Figure 4b showed different profiles compared to non-stabilized ones. Interestingly, the profile of cross-linked P(VDF-HFP)/PEGDA/IL-L indicates almost complete absence of phase transitions in the evaluated temperature range. This finding may be due to the increased thermal resistance of the stabilized membrane as a result of the formation of polar bonds between the ionic liquid species and the polymer network. Such a high thermal stability was already observed in the TGA curve, where no mass loss was observed before the occurrence of thermal decomposition at about 400 °C (see Figure 3b).

**Figure 5:**
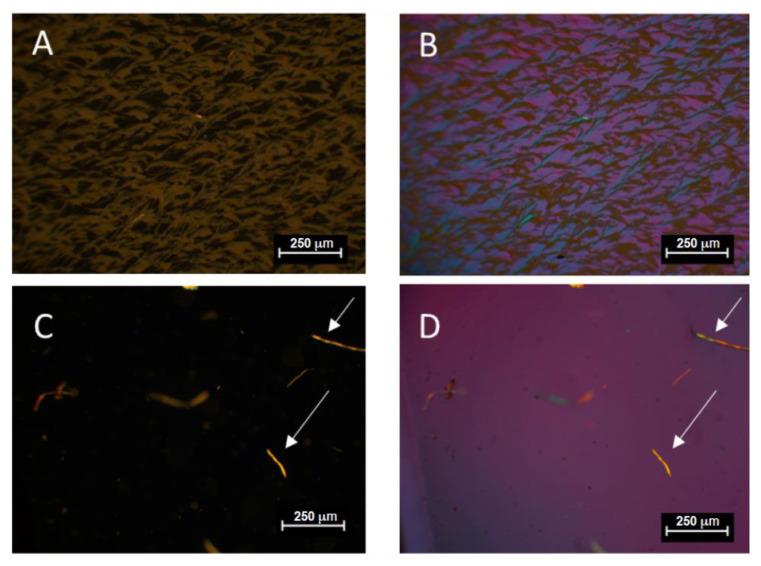
POM images obtained without (**A**,**C**) and with (**B**,**D**) crossed polarizers of P(VDF-HFP)/IL composite membranes: (**A**,**B**) cross-linked pure P(VDF-HFP) film; (**C**,**D**) cross-linked P(VDF-HFP)/IL-L film (scale bar 250 μm).

Polarized optical microscope (POM) (SEM presented in Figure S2, ESI section) images of the cross-linked pure P(VDF-HFP) polymer films and doped with IL composite ones were taken using a POM at 100× magnification in order to highlight the effect of IL doping on the cross-linked polymeric texture (Figure 5). The images of a stabilized non-doped pure polymer membrane show the successful preparation of films with a rough structure with cracks achieved by P(VDF-HFP) dissolution in a mixture of solvent/non-solvent (acetone/ethanol) (a,b images) after their evaporation at the end of the deposition–solution casting technique and the subsequent cross-linking by polymerization of PEGDA. Acetone is a good solvent for the polymer and is more volatile than ethanol (non-solvent for P(VDF-HFP)). In the course of solvent evaporation, the polymer solution becomes richer in ethanol and the polymer precipitates in the non-solvent phase. As a result, the rough structure of the polymer matrix is formed after ethanol evaporation (Figure 5A,B and Figure S2a). As observed in the images, uneven, granular surfaces with bright and dark fields of lamellar distribution assume the semi crystalline nature of the film. The micrographs of IL containing membranes show the appearance of large, homogeneous smooth area as the dark background as a result of interaction between the polymer and salt. Upon incorporation of ionic liquid into the cracks, the appearance of “luminous” areas in POM images is observed (Figure 5C,D) (bright dots in SEM image, Figure S2b). The observed increase by two orders of magnitude in the conductivity of these films is also a confirmation of the stated assumption of the inclusion of the ionic liquid into the cracks of the polymer matrix localized in them after cross-linking. The cross-linking points provided by PEGDA impart a network structure to the polymer matrix thus improving its mechanical stability. On the other hand, such a structure provides sufficient incorporation of the IL into the polymer matrix taking place simultaneously with the polymerization process. The latter could explain the observed higher thermal stability (see the above paragraph) of the crosslinked doped membrane and its improved conductivity (see Table 1 placed in electrochemical section) which may be attributed to the polar bonding occurring between the oxygen containing functional group of the polymer matrix and the cation of the IL as it was already supposed by others [27,28,29].

**Figure 6:**
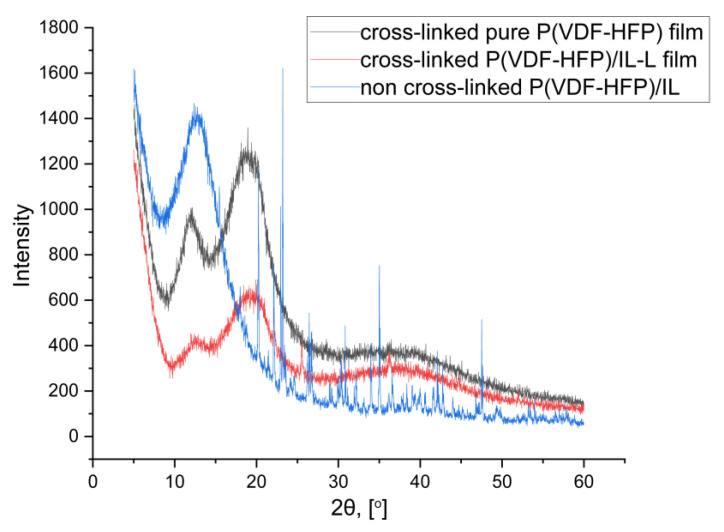
XRD profile of P(VDF-HFP)/IL-L (blue curve), cross-linked pure polymer (black curve) and cross-linked doped with IL (red curve) films.

The XRD pattern (Figure 6) of non-cross-linked film shows many sharp diffraction peaks belonging to the crystal structure of IL. The broad peak at 12° is relative to the sample holder, which is made of poly(methyl methacrylate) PMMA pad. In contrast, in the pattern of the cross-linked films, a new broad peak appears at 20° corresponding to the (020) crystalline peaks of P(VDF-HFP) [1]. The presence of this peak indicates the semi-crystalline nature of the polymer in which crystalline regions are mixed with the amorphous phase. The selected procedure of stabilized film preparation by solution casting of the polymer, dissolved in a mixture of solvent/non-solvent and subsequent cross-linking in the presence of IL, initiates some arrangement of the macromolecules of P(VDF-HFP). Further, the inclusion of IL in the film broadens the existing peak at 20° and confirms the complete blending of IL with the PVDF-HFP matrix at the molecular level, which explains the observed enhancement of ionic conductivity by two orders of magnitude (see Table 1) of the cross-linked P(VDF-HFP)/IL-L film compared to non-crosslinked doped polymer films.

The data from XRD analysis are in good agreement with the observed different morphologies of the POM images of P(VDF-HFP)/IL-L and cross-linked IL-doped films. The inclusion of IL leads to formation of homogeneous texture with increased surface roughness into the polymer matrix, which assumes better IL distribution in the membrane, prevents the polymer chains from crystallizing and thus increases the film’s amorphousness.

The data from XRD analysis are also consistent with the observed different morphology of the POM images of P(VDF-HFP)/IL-L and cross-linked IL-doped films. The inclusion of IL leads to formation of homogeneous texture into the polymer matrix, which assumes better IL distribution in the membrane, prevents the polymer chains from crystallizing and thus increases the film amorphousness.

Last but not least, it should be noted that cross-linking of the membrane leads to an increase in its density, thus lowering the swelling ability of the polymer matrix in regard to IL. Contrary, when IL uptake increases in the gel polymer matrix the conductivity is also expected to increase; thus, compensating for the decreased swelling capacity as has been speculated by others [13].

### 2.1. Electrochemical Results

The preparation of membranes with an IL-L content in BMPTFSI/P(VDF-HFP) above 10/1 (wt/wt) results in a film with poor mechanical properties. Films with higher IL content can be prepared using EMIMI (IL-S), but their conductivity decreases with increasing its weight content (Table 1). On the other hand, they are unstable and tend to release a certain amount of ionic liquid, but after crosslinking these films become brittle. This provided us with a reason to investigate P(VDF-HFP)/IL-L and P(VDF-HFP)/PEGDA/IL-L gel polymer films as separators in a supercapacitor cell.

#### 2.1.1. P(VDF-HFP)/IL-L (1-Buthyl-1-methylpyrrolidinium Bis(trifloromethylsulfonyl)imide) Film

**Figure 7:**
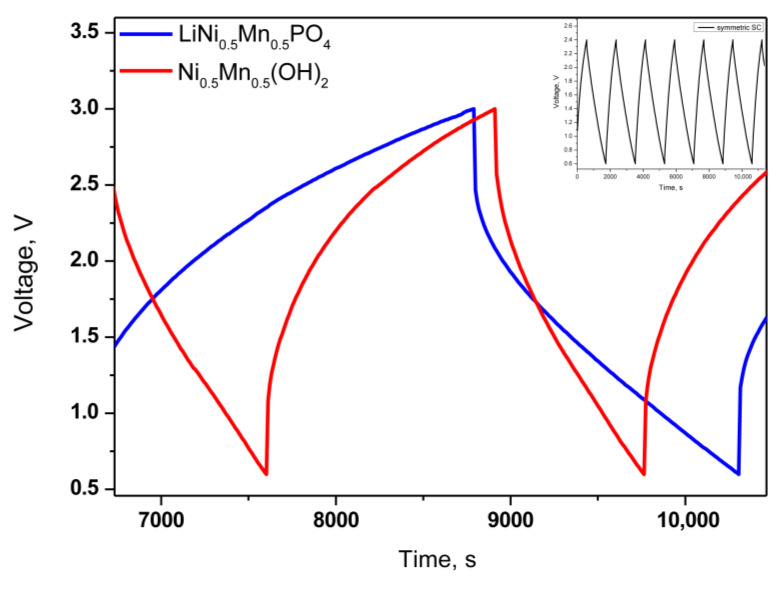
Galvanostatic charge–discharge curves of supercapacitor cells with different composite and P(VDF-HFP)/IL-L–film at current rate 60 mAg^−1^, inset figure—symmetric SC.

The obtained gel film P(VDF-HFP)/IL-L is used as separator in symmetric and hybrid supercapacitors, previously soaked in IL and then 20 µL IL was added to the cell. The electrochemical cells with mixed hydroxides and phosphate and P(VDF-HFP)/IL-L film as separator in voltage windows up to 3.0 V display charge/discharge curves whose shapes are typical for hybrid supercapacitor behavior (Figure 7). The symmetric device (inset figure) shows the ideal EDL electrochemical response.

**Figure 8:**
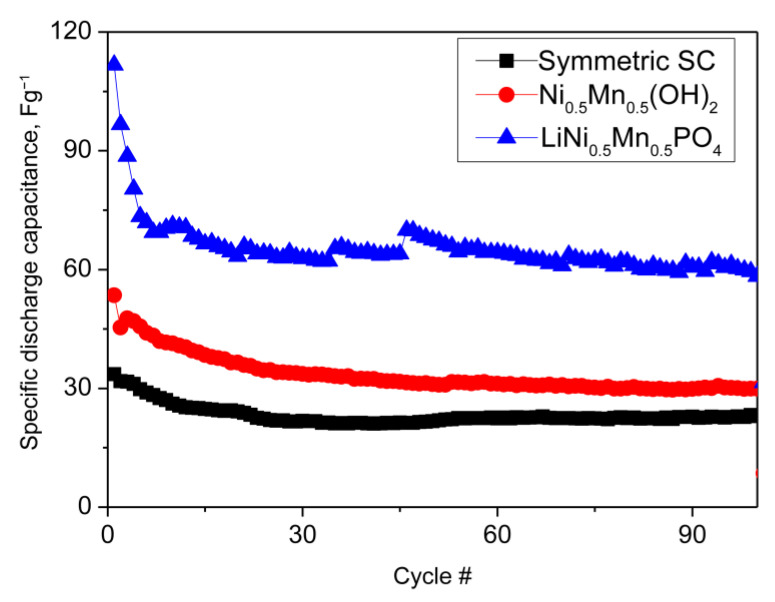
Discharge capacitance as a function of the cycle number of symmetric and hybrid supercapacitors with P(VDF-HFP)/IL-L–film, soaked in IL electrolyte.

To more precisely compare the electrochemical performance of the P(VDF-HFP)/IL-L film used as a separator, the electrochemical cells were cycled at DC for 80 cycles at different current loads (Figure 8). According to the results, the hybrid SC demonstrated higher capacitance than the symmetrical one. The highest value is provided by the olivine composite electrode. These results indicate that the obtained gel films are suitable for use in SC. It should be noted that N–OH shows a lower capacitance than that of the phosphate electrode, which is in contrast to our previous studies in KOH and to its many-times-higher surface area than that of olivine [30].

For the interpretation of the strange result obtained at first sight, it should be kept in mind that the high active surface of the electrode material is not the only answer for the high capacity. The factors are many and interrelated. Here, on one hand, the morphology of the electrode material must be taken into account, as well as the physicochemical characteristics of the polymer film providing ion transport in the cell. This shows the complex characteristics of energy storage in these systems, including the capacitive and Faraday reactions taking place in the hybrid systems.

The stability of the resulting gel polymer film was proven by conducting long-term tests for 5000 cycles at a current load of 60 mA g^−1^. For example, for symmetrical SC, the discharge capacitance loss does not exceed 15% after 5000 charge/discharge cycles and the current efficiency is above 95% (Figure S3). The results show that the prepared polymer network film can be successfully used as a separator in a cell with an ionic liquid electrolyte.

#### 2.1.2. Crosslinked P(VDF-HFP)/PEGDA/IL-L Film

The impedance measurements performed show that the cross-linked films possess higher conductivity compared to the non-stabilized ones and do not lose the ionic liquid content incorporated in them. These properties, together with their high mechanical stability, are a prerequisite for possible application of these films as both separator and electrolyte in supercapacitor cells.

**Figure 9:**
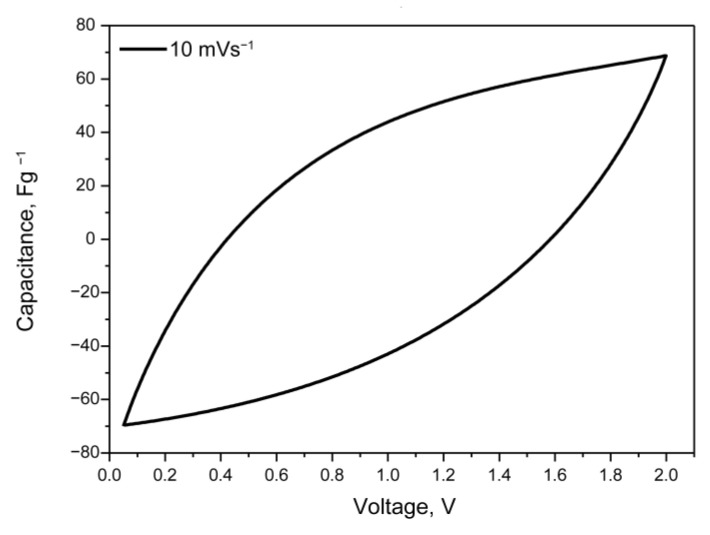
Cyclic voltammetry curve of symmetric supercapacitor cell with cross-linked film P(VDF-HFP)/PEGDA/IL-L at scan rate 10 mVs^−1^.

Symmetric supercapacitor cells with film P(VDF-HFP)/PEGDA/IL-L as separator and electrolyte were assembled (without soaking and addition of IL) and tested using cyclic voltammetry and galvanostatic charge/discharge measurements in a two-electrode cell with scan rate varying from 1 to 100 mVs^−1^. The CV curve in a voltage window up to 2.0 V are generally typical for symmetric supercapacitor systems, although the capacitive behavior is not very pronounced (Figure 9)

The thermal stability of the polymer gel films is shown by the thermogravimetric analysis (TGA) data in Figure 3. In addition, the electrochemical stability window (ESW) of traditional electrolytes decreases rapidly with increasing temperature, while that of ionic liquids is wider, they are non-volatile and non-flammable and are promising electrolytes for EDLC operations at high temperatures [14].

**Figure 10:**
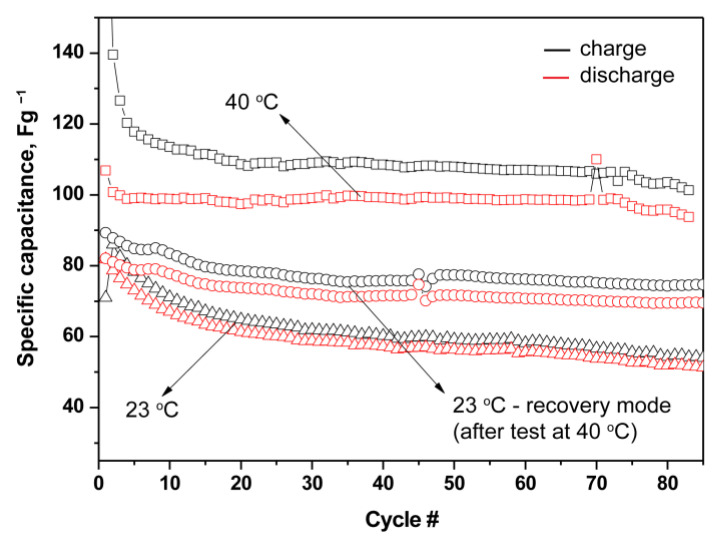
Capacitance as a function of the cycle number of symmetric supercapacitors with cross-linked film P(VDF-HFP)/PEGDA/IL-L, at room temperature and 40 °C.

This provided us with the reason to investigate the capacitive properties of SC with crosslinked film at elevated temperature. In Figure 10, the results of galvanostatic charge-discharge curves at room temperature (23 °C) and at 40 °C are presented.

As expected, with increasing operating temperatures, at elevated temperature, the SCs show a higher discharge capacitance (from 57 to 99 Fg^−1^ at the 40th charge/discharge cycle, i.e., about a 40% increase), although the current efficiency decreases slightly (from 94% to 91%). Furthermore, when the temperature is subsequently lowered again to room temperature, the specific capacitance is maintained and even shows higher values than the initial ones, with the current efficiency decreasing by about 1%.

**Figure 11:**
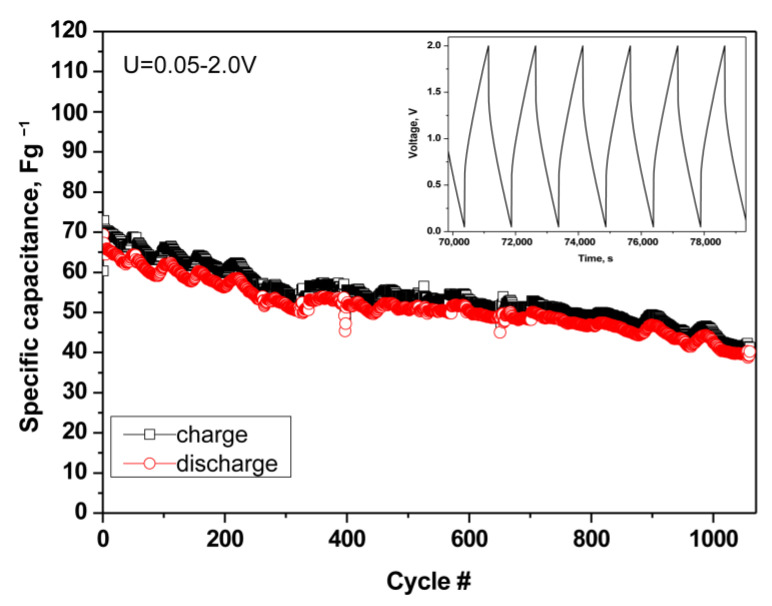
Long-term test of symmetric SC cell with cross-linked film P(VDF-HFP)/PEGDA/IL-L at a current load of 60 mA g^−1^.

To verify the stability of the P(VDF-HFP)/PEGDA/IL-L film, the supercapacitor was subjected to long-term tests for more than 1000 charge and discharge cycles (Figure 11).

After 1100 cycles, the symmetric SC delivered good cycling stability and high current efficiency (around 96–97%), which can be directly associated with the chemical stability of the electrodes and the mechanical one of the gel polymer film. It should be noted that the charge–discharge curves exhibit triangular-shaped profiles, which are typical for the capacitive storage mechanism (i.e., electrical double-layer).

These results confirm that the supercapacitors assembled with ILQSE are suitable for use as separator and electrolyte in SC systems. Further research is needed to develop a high-temperature solid-state supercapacitor with enhanced capacitance characteristics, as well a hybrid one.

## 3. Conclusions

Various IL-based GPE membranes have been prepared using the solution casting technique. Physicochemical characterization shows that they are mechanically and thermally stable. Their additional crosslinking results in stabilized structures displaying up to two orders of magnitude higher ionic conductivity. Electrochemical tests carried out with selected films showed that they are suitable for use in SCs, replacing the separator in them with no need to use an electrolyte. Additionally, the developed symmetric cross-linked film supercapacitor exhibits very stable characteristics and is promising for the development of high-temperature solid-state supercapacitors with high capacitance characteristics as well as high energy and power densities.

## 4. Materials and Methods

### 4.1. Materials

All chemicals were purchased from Sigma-Aldrich, St. Louis, MO, United States. Poly(ethylene glycol) diacrylate (PEGDA) (*M*_n_ = 575 g/mol) was passed through a column containing neutral aluminum oxide. Azobisisobutyronitrile (AIBN) was recrystallized from methanol. Poly(vinylidene fluoride-co-hexafluoropropylene) P(VDF-HFP) (pellets, M_w_ = 236,000, MWD = 2.8), 1-butyl-1-methylpyrrolidinium bis(trifluoromethylsulfonyl)imide (BMPTFSI) (>99%), 1-ethyl-3-methylimidazolium iodide (EMIMI) (97%), acetone (>99.5) and ethanol (96%) were used as received.

#### 4.1.1. GPE Films Preparation and Cross-Linking

##### Preparation of Ionic Liquid-Based Gel Polymer Electrolytes (P(VDF-HFP)/IL-L (S))

The ionic liquid-based gel polymer electrolytes were prepared by applying the solution-cast technique. Firstly, 0.250 g of P(VDF-HFP) was dissolved in 5 mL of acetone by continuous stirring for 1 h on a magnetic stirrer. Then, 1 g of ionic liquid 1-butyl-1-methylpyrrolidinium bis(trifluoromethylsulfonyl)imide (BMPTFSI) (IL-L) or 1-ethyl-3-methylimidazolium iodide (EMIMI) (IL-S) was added to the polymer solution and stirred for another 1 h. The obtained solutions were transferred into glass Petri dishes and a slow evaporation of the solvent was allowed. Finally, flexible films were obtained. Separately, the film with 0.025 g KI as redox-additive per gram of P(VDF-HFP) was obtained. For the further experiments, films with different weight ratios between P(VPF-HFP) and the corresponding IL were prepared: P(VDF-HFP)/BMPTFSI = 1/4 (wt/wt) or 1/10 (wt/wt) and P(VDF-HFP)/EMIMI = 1/20 (wt/wt), 1/10 (wt/wt) or 1/4 (wt/wt).

##### Preparation of Gel Polymer Electrolytes Network (P(VDF-HFP)/PEGDA/IL-L (S))

Initially, 0.5 g of P(VDF-HFP) was dissolved in 7.5 mL of acetone. Then, 2 g IL (L or S) (BMPTFSI or EMIMI) was added and the mixture was stirred for 30 min until complete dissolution. Finally, 0.3 g PEGDA (3/5 wt/wt PEGDA/PVDF), 0.1 g AIBN (1 wt%) and 2.5 mL of ethanol were added (the indicated weight precents are towards all components in the mixture including the solvents). The obtained viscous solution was cast into glass Petri dishes. The polymer membranes were obtained after slow evaporation of acetone and ethanol at room temperature for 12 h. Finally, the membranes were heated in a vacuum oven at 80 °C for 12 h in order to initiate and perform the cross-linking reaction.

### 4.2. Methods

#### 4.2.1. GPEs Films Characterization

##### Membrane Conductivity

A Fumatech MK3 measurement cell equipped with a potentiostat/galvanostat AutoLab model PGSTAT204FRA32M, Metrohm AG, Herisau, Switzerland was used to perform impedance measurements. The membrane conductivity was determined by four-probe impedance measurements in the frequency range of 0.1 × 10^−6^ Hz with a 10 mV signal at room temperature and humidity. The membrane having ~1.5 cm width was placed on top of the four platinum wire electrodes which were placed on a Teflon disc with a distance of 1 cm between them. The ionic conductivity was calculated according to equation *σ = L/R.A* (1), where: σ is the ionic conductivity (in S.cm^−1^), *L* is the distance between the electrodes (cm), *A* is the membrane section area (in cm^2^) and *R* is the impedance of the membrane (in ohms).

##### Thermal Analyses

Thermal analyses were carried out using a Perkin ElmerDSC-8500, WA, USA differential scanning calorimeter (DSC) system in the temperature range −60 to 180 °C at a heating rate of 10 °C min^−1^ and using a Perkin Elmer 4000 thermogravimetric analysis (TGA) apparatus heated from 40 to 800 °C at a heating rate of 15 °C min^−1^ under continuous purging of nitrogen.

##### FT-IR

Infra-red absorption spectra were measured using a Shimadzu 8400S Affinity FTIR spectrometer at a resolution of 4 cm^−1^ in the frequency range from 600 to 4000 cm^−1^. The Morphology of the Electrode Surface Was Visualized with a Leica DMLP Optical Microscope, Leica Microsystems GmbH, Wetzlar, Germany.

##### Wide-Angle X-ray Diffraction (WAXD)

Wide-angle X-ray diffraction (WAXD) scans were obtained using a Bruker D8 Advance ECO diffractometer Bruker AXS GmbH, Karlsruhe, Germany, operating at 40 kV and 25 mA in Bragg–Brentano geometry with Ni-filtered Cu K_α_ radiation and a LynxEye-XE detector over the 2θ range of 5–60°, with a scanning rate of 0.02°·s^−1^

#### 4.2.2. Electrochemical Tests

The electrochemical performances of the obtained polymer films used as separators and electrolyte membranes in supercapacitor cells were examined in symmetric and hybrid two-electrode cells. Assembly of cells with non-cross-linked film was performed in a dry argon box where the polymer film was soaked with an ionic liquid, which was also added to the cell itself. When cross-linked film is used, it is placed dry in the supercapacitor cell, which is assembled in air without adding ionic liquid to it.

An activated carbon commercial product (Kuraray Europe GmbH) was used. Mixed hydroxide with layered structure (β-type Ni_0.5_Mn_0.5_(OH)_2_) and phosphate with olivine structure (LiNi_0.5_Mn_0.5_PO_4_) were used as composite electrode materials in hybrid supercapacitors. These multiphase compounds were obtained by specific synthetic procedures and showed high capacitive characteristics and good cycling stability in alkaline electrolytes due to the synergistic effect of Ni^2+^ and Mn^2+^ ions [30]. Poly(vinylidene fluoride) PVDF (Sigma Aldrich, St. Louis, MO, USA) (10 wt%) and graphite ABG 1005 EG1 (10 wt%) were used as binder and conductive additive.

The capacitor cells were subjected to cyclic voltammetry (CV) measurements using a Multi PalmSense system (model 4, The Netherlands) and galvanostatic charge–discharge tests were performed using an Arbin Instrument System BT-2000, Arbin Instruments-Beijing, China

The capacitance (F g^−1^) was calculated from the charge–discharge curves using the following the equation [31,32]: *C* = (*I* × Δ*t*)/(*m* × Δ*V*)(2), where: *I* (A) is the discharge current, Δ*t* (s) is the mass of the active material and *m* (g) and Δ*V* (*V*) is the voltage window.

## Figures and Tables

**Table 1:** Ionic conductivity of obtained polymer films.

Film	P(VDF-HFP)	P(VDF-HFP)/IL-L 1/4 (wt/wt)	P(VDF-HFP)/IL-L 1/10 (wt/wt)	P(VDF-HFP)/IL-S 1/4 (wt/wt)	P(VDF-HFP)/IL-S 1/10 (wt/wt)	P(VDF-HFP)//PEGDA/IL-L 1/10 (wt/wt)
Conductivity, mS/cm	5.0 × 10^−9^	1.1 × 10^−3^	1.5 × 10^−3^	2.5 × 10^−3^	0.5 × 10^−3^	8.5 × 10^−1^

## Data Availability

The data that support the findings of this study are available within the article.

## Supplementary Materials

The following supporting information can be downloaded at: https://www.mdpi.com/article/10.3390/gels9040338/s1, Table S1: Assignment of FTIR spectral bands of pure PVdF-HFP and BMPTFSI [1,17]; Figure S1: FTIR spectra of pure P(VDF-HFP), IL-L and P(VDF-HFP)/IL-L; Figure S2: SEM images of PVDF-HFP/IL composite membranes: (a) cross-linked pure P(VDF-HFP) and (b) cross-linked P(VDF-HFP)/IL-L; Figure S3: Long-term test of symmetric SC cell with P(VDF-HFP)/IL-L film at a current load of 60 mA g^−1^.
