# Supplementary material for: Flexible Polymer-Ionic Liquid Films for Supercapacitor Applications
Source: Gels. 2023 Apr 16;9(4):338. doi: 10.3390/gels9040338 (PMC10137811; doi:10.3390/gels9040338)
Supplement: Supplementary file 1 [file gels-09-00338-s001.zip › gels-2298827-supplementary.pdf]

gels

Article

Flexible Polymer-Ionic Liquid Films for Supercapacitor Applications

Christo Novakov [1,*], Radostina Kalinova [1], SvetlanaVeleva [2], Filip Ublekov [1], Ivaylo Dimitrov [1] and Antonia Stoyanova [2,*]

1 Institute of Polymers, Bulgarian Academy of Sciences, Sofia, Acad. G. Bonchev Str., 103, 1113 Sofia, Bulgaria
2 Institute of Electrochemistry and Energy Systems, Bulgarian Academy of Sciences, Acad. G. Bonchev Str., 10, 1113 Sofia, Bulgaria
* Correspondence: hnovakov@polymer.bas.bg (C.N.); antonia.stoyanova@iees.bas.bg (A.S.)

Supporting Information

Table S1. Assignment of FTIR spectral bands of pure PVdF-HFP and BMPTFSI [1,17].

	IR (cm^{-1})	Assignment
**PVdF-HFP**	489	Bending and wagging of the CF_2 group
	613	C-F_2 bending and CCC skeletal vibration
	762	CH_2 rocking vibration
	795	CF_3 stretching vibration
	840	CH_2 rocking
	974	C-F stretching
	1066	Vibration of crystalline phase
	1181	C-C band stretching
**BMPTFSI**	510, 571	CF_3 asymmetric bending
	598	bending CH_3(N)CN stretching
	654	C-H vibrational mode of cyclic BMP^+
	740	S-N stretching mode
	793	C-C-H bending
	1051	Asymmetric S-N-S stretching
	1132	C-SO_2-N bending
	1349	Asymmetric SO_2 stretching
	1469	CH_2, CH_3

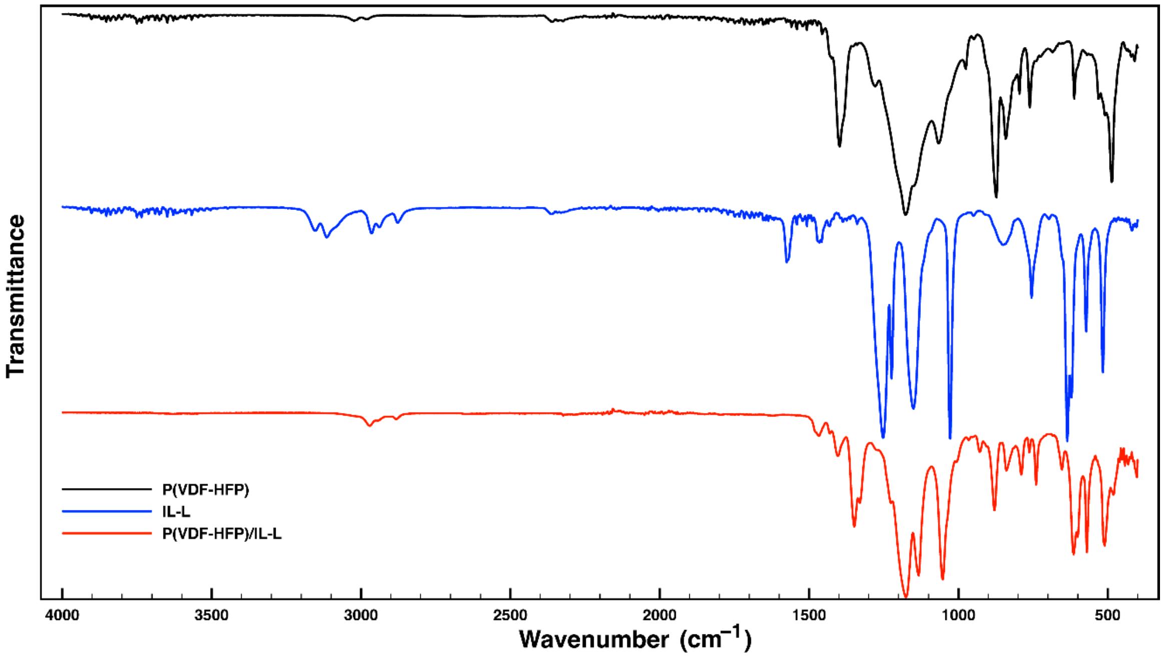

Figure S1. FTIR spectra of pure P(VDF-HFP), IL-L and P(VDF-HFP)/IL- L.

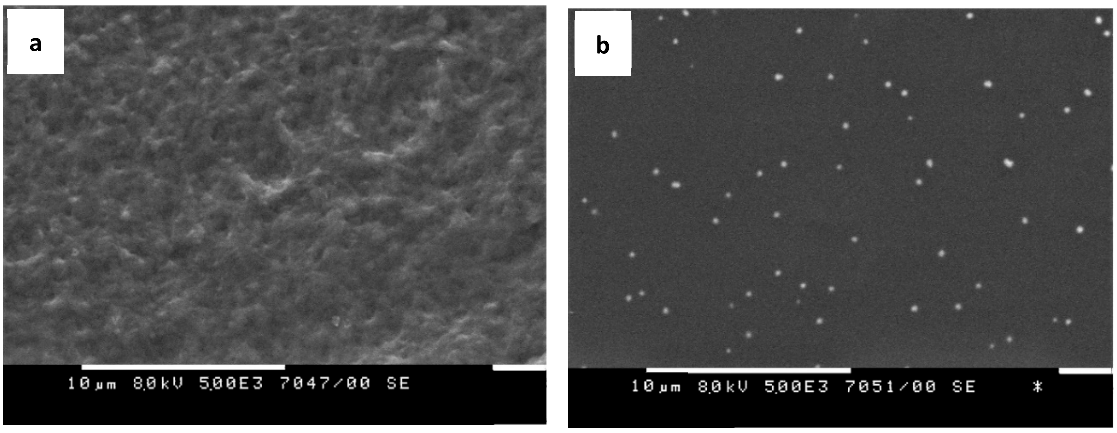

Figure S2. SEM images of PVDF-HFP/IL composite membranes: a) cross-linked pure P(VDF-HFP) and b) cross-linked P(VDF-HFP)/IL-L.

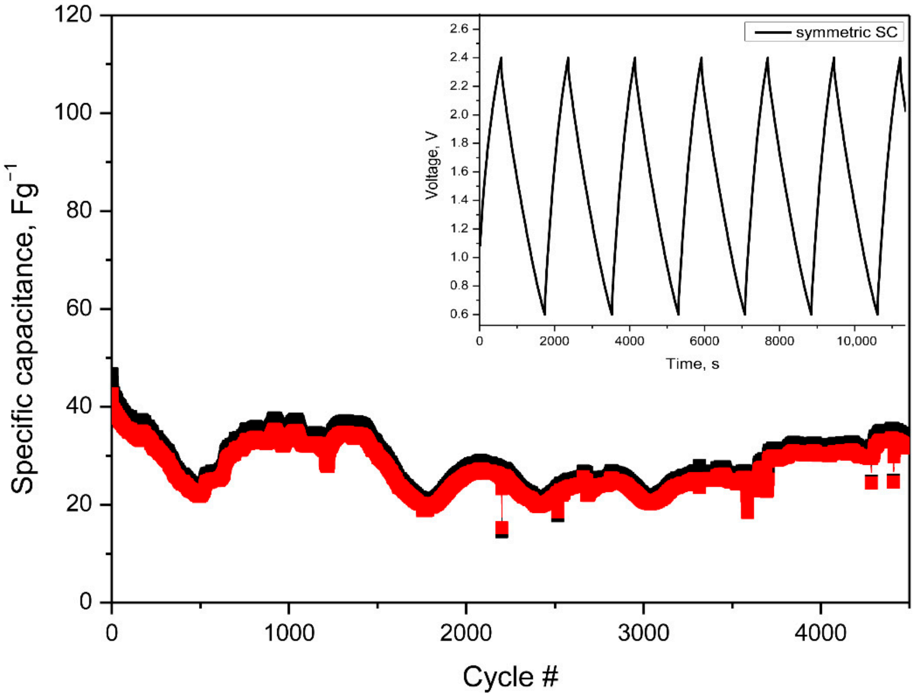

Figure S3. Long-term test of symmetric SC cell with P(VDF-HFP)/ IL-L film at a current load of 60 mA $g^{-1.}$
